# Infant growth and body composition from birth to 24 months: are infants developing the same?

**DOI:** 10.1038/s41430-023-01386-5

**Published:** 2024-01-03

**Authors:** Shane A. Norris, Lukhanyo H. Nyati, Alexia Murphy-Alford, Nishani Lucas, Ina S. Santos, Caroline S. Costa, Rebecca Kuriyan, V. Pujitha Wickranasinghe, Shabina Ariff, Sisitha Jayasinghe, Anura V. Kurpad, Leila Cheikh Ismail, Andrew P. Hills, Multi-center Infant Body Composition Reference Study (MIBCRS), Lukhanyo H. Nyati, Nishani Lucas, Ina S. Santos, Caroline S. Costa, Rebecca Kuriyan, V. Pujitha Wickranasinghe, Shabina Ariff, Sisitha Jayasinghe, Anura V. Kurpad, Andrew P. Hills, Shane Norris, Alexia J. Murphy-Alford, Leila C. Ismail, Tanvir Ahmad, Kiran D. K. Ahuja, Jeff M. Beckett, Renata M. Bielemann, Nuala M. Byrne, Laila Charania, Michele P. Christian, Priscilla J. Divya, Anne Hanley, Manoja P. Herath, Pulani Lanerolle, Cornelia Loechl, Najat Moktar, Upul Senerath, Christine Slater, Sajid Soofi, Steven J. Street, Neiva C. J. Valle, Ayesha Yameen

**Affiliations:** 1 SAMRC Developmental Pathways for Health Research Unit, Department of Pediatrics, University of the Witwatersrand, Johannesburg, South Africa; 2 School of Human Development and Health, University of Southampton, Southampton, UK; 3 Interprofessional Education Unit, Faculty of Community and Health Sciences, University of the Western Cape, Cape Town, South Africa; 4 International Atomic Energy Agency, Vienna, Austria; 5 Department of Paediatrics, Faculty of Medicine, University of Colombo, Colombo, Sri Lanka; 6 Federal University of Pelotas, Pelotas, Brazil; 7 Division of Nutrition, St John’s Research Institute, Bengaluru, India; 8 Dept Pediatrics & Child Health, The Aga Khan University, Karachi, Pakistan; 9 University of Tasmania, Hobart, TAS Australia; 10 University of Sharjah, Sharjah, United Arab Emirates; 11 Nuffield Department of Women’s & Reproductive Health, University of Oxford, Oxford, UK; 12 Isotope Application Division, Islamabad, Pakistan

**Keywords:** Epidemiology, Risk factors

## Abstract

**Background:**

Given the importance of infancy for establishing growth trajectories, with later-life health consequences, we investigated longitudinal body composition among infants from six economically and ethnically diverse countries.

**Methods:**

We recruited mother-infant dyads using the WHO Multicenter Growth Reference Study criteria. We measured fat-free mass (FFM) in 1393 (49% female) infants from birth to 6 months of age (Australia, India, and South Africa; *n* = 468), 3–24 months of age (Brazil, Pakistan, South Africa, and Sri Lanka; *n* = 925), and derived fat mass (FM), fat mass index (FMI), and fat-free mass index (FFMI). Height-for-age (HAZ), weight-for-age (WAZ), and weight-for-length (WHZ) Z-scores were computed. Sex differences were assessed using a t-test, and country differences using a one-way analysis of covariance. We further compared subsamples of children with average (−0.25 > HAZ < +0.25), below-average (≤−0.25) and above-average (≥+0.25) HAZ.

**Results:**

HAZ performed well between 0 and 6 months, but less so between 3 and 24 months. The stunting prevalence peaked at 10.3% for boys and 7.8% for girls, at 24 months. By 24 months, girls had greater FMI (10%) than boys. There were significant differences in FFM (both sexes in all countries) and FM (Brazilian boys, Pakistani and South African girls) by 24 months of age between infants with average, above-average, and below-average HAZ.

**Conclusion:**

In a multi-country sample representing more ideal maternal conditions, body composition was heterogeneous even among infants who exhibited ideal length. Having a mean HAZ close to the median of the WHO standard for length reduced FFM between-country heterogeneity but not FM, suggesting that other factors may influence adiposity.

## Introduction

A good start to life is critical to offset later risks including lower school achievement, reduced adult income, non-communicable disease susceptibility, and intergenerational risk [1]. Growth failure and/or rapid weight gain in early life is also associated with an array of adverse health effects in later life [2–4]. The WHO Growth Standards have provided better means of interpreting length and weight assessments independent of where the child is born. Along with normative data for foetal growth [5], the child growth standards form part of recent advances in monitoring child development. Normative data for body composition have been described as the next logical level of advancement [6]. The Multi-center Infant Body Composition Reference Study (MIBCRS) has come close to achieving this by developing reference charts for body composition using a multi-ethnic sample, to represent a suitable reference for body composition gain [7].

Data from the MIBCRS emphasise the important role of context [8], home environment and feeding practices [9] in shaping body composition. However, the performance of body composition relative to ideal growth, which is the gold standard for assessing child development in both clinical and epidemiological settings, remains unclear. While anthropometric measurements may provide better reproducibility and are easy to obtain, body composition assessments may provide a more precise picture of the biological response to the interplay of genetic, nutrition, and environmental effects than measures of body size. A better understanding of infant body composition patterns in addition to linear growth may present possible intervention opportunities to reduce the obesity risk in infancy and childhood, and possibly offset the risk of non-communicable disease in adulthood. Therefore, our aim was to investigate longitudinal sex and country differences in infant body composition relative to ideal growth in six countries under conditions that are more likely to facilitate improved linear growth. We hypothesised that children born to mothers selected accordingly to the WHO MGRS [10] will track the WHO growth standards median and have similar FMI and FFMI during infancy irrespective of which country they were born in.

## Methods

### Study settings

Data were from the Multi-center Infant Body Composition Reference Study (MIBCRS), a longitudinal, multinational study that followed infants from birth to 24 months of age in lower-middle (India, Pakistan, and Sri Lanka), upper-middle (Brazil and South Africa), and high-income (Australia) countries. The study complied with the International Ethical Guidelines for Biomedical Research Involving Human Subjects, and each participating country-site obtained ethical approval from its respective review committee. Written informed consent was obtained from mothers enrolled in the study. Data collection commenced in 2013 and was completed by December 2019.

### Participant recruitment

Participant infants were enrolled at birth at the Launceston General Hospital, Tasmania (Australia), at the five hospitals with a maternity ward in Pelotas (Brazil), at St John’s Medical College Hospital, Bangalore (India), at Aga Khan University Hospital in Karachi (Pakistan), Chris Hani Baragwanath Academic Hospital in Johannesburg (South Africa), and at the University Unit of the De Soysa Hospital for Women, in Colombo (Sri Lanka). We recruited 1467 mother-infant dyads (48.7% girls); 476 infants in the sub-study using air-displacement plethysmography (ADP; 2089 data points) to determine body composition at birth, 1, 2, 3, 4 and 6 months of age, and 991 infants in the sub-study using the deuterium dilution technique (DD; 4503 data points) at 3, 6, 9, 12, 18 and 24 months of age. We excluded participants with flagged values (biologically implausible Z-scores and low- or high enrichment) and included 925 children (3708 observations) with data using the DD technique, and 468 children (1899 observations) using ADP. The sample size was calculated for the study sites to have a power of 90% to detect fat mass (FM) and fat-free mass (FFM) for boys and girls less than one standard deviation away from a reference study, that found a mean FM of 3.10 ± 0.5 kg and 3.05 ± 0.46 kg, and mean FFM 9.13 ± 1.06 kg and 8.99 ± 1.1 kg for boys and girls, respectively, which resulted in an estimation of 100 participants per site, 300 for birth to 6 months, and 400 for the 3 to 24 months cohorts [11].

### Screening

We recruited women based on: a healthy singleton term infants born within 37 and 42 weeks of gestation, being 18 years or older, a non-smoker with at least secondary school education level, and willing to breastfeed for at least up to 6 months. Infants were to be without morbidity and congenital abnormalities. Infants with significant morbidity such as cardiorespiratory illnesses and congenital abnormalities which might affect infant growth, and mothers living with HIV/AIDS were excluded. Our criteria were consistent with those used in the WHO Multicenter Growth Reference Study (MGRS) and the INTERGROWTH-21^st^ Project [10]. Maternal demography, obstetric history, and education were recorded using questionnaires. Breastfeeding and feeding practices were collected from birth in the birth to 6 months cohort and from the 3-month follow-up in the 3 to 24 months cohort. Infants were classified as being exclusively breastfed (EBF) if they did not consume semi-solid or solid foods and fluids. Questions on consumption on meat, poultry and egg were not asked in Sri Lanka. Excluding these questions in the computation of EBF did not change the prevalence in all countries. Consumption of pure water also did not significantly influence the prevalence of EBF in all countries except South Africa.

### Anthropometry

Standardised protocols for anthropometry were developed based on the WHO-MGRS protocol. Newborns were weighed and length measured no later than 24 h after birth. Infant weight and length measurements took place at 1, 2, 3, 4, 6, 9, 12, 18, and 24 months. Weight was measured naked, using a pediatric electronic scale (Seca 376; Hamburg, Germany), accurate to the nearest 5 g. Length was measured using a Harpenden infantometer (300–1100 mm, accurate to 1 mm; Holtain Ltd, Crymych, Wales, UK) in all countries, except India and Sri Lanka, where they used the SECA 417 infantometer.

### Air-displacement plethysmography (ADP)

In the birth to 6-month cohort, body composition was assessed by ADP (PEA POD, Software version 3.5.0, 201, COSMED USA) within 2–3 days of birth, and at 1, 2, 3, 4, 6 months employing standard procedures [12]. Total body density was calculated as the ratio of weight (kg) and the measured body volume (L) and was used to calculate the proportions of FM and FFM using assumed densities (0.9007 and 1.063 kg/L for FM and FFM, respectively).

### Deuterium dilution (DD) technique

In the 3-to-24-month cohort, body composition was collected using DD at 3, 6, 9, 12, 18, and 24 months of age. Total body water (TBW) is predicted from the dilution of a known quantity of deuterium oxide (D_2_O) in body water, which is evenly distributed throughout the body within a few hours and can be sampled from saliva. From 3 to 9 months of age, all infants received an oral dose of 1 g sterile D_2_O (99.8 atom % ^2^H). From 12 to 24 months, the dose was increased to 1.5 g. The D_2_O was administered to the infant using a 3 mL syringe. The syringe was filled with the required amount of D_2_O and weighed before and after administration to determine the weight of D_2_O consumed. Saliva was sampled before D_2_O administration (baseline) and 2.5 and 3 h after administration. The baseline saliva sample was collected at least 15 min after the last feed, to ensure that no waste milk or other food was in the child’s mouth. Saliva samples were collected by moving swabs wrapped in cotton inside the child’s mouth until they were soaked. This procedure was repeated until 1 mL of saliva had been collected. If any D_2_O was regurgitated, the protocol was interrupted, and the mother was invited to return in seven days. Saliva samples were stored in a −20 °C freezer until analysis. The enrichment of deuterium in saliva was measured by isotope ratio mass spectrometry (IRMS) or by Fourier transform infrared (FTIR) spectrometer (Agilent 4500 Series). TBW was calculated using the weight of D_2_O consumed, the enrichment of the deuterium in the dose, and the enrichment of deuterium in the 3-h saliva sample, with a small correction (4.1%) for non-aqueous exchange of deuterium. FFM was estimated by dividing by an age-related constant for the hydration of FFM, and FM was the difference between body weight and FFM.

### Data management and analyses

Data were captured on the REDCap system [13]. The WHO-2006 child growth standards for children from birth to 5 years were used to generate height-for-age (HAZ), weight-for-age (WAZ), and weight-for-length (WHZ) Z-scores in R, flagging biologically implausible values [14, 15]. Additional cleaning was performed with box plots and the plot clean function in the SITAR package (version 1.0.9) in R [16]. Stunting, underweight and wasting were defined as a Z-score of less than minus two (−2) of the median HAZ, WAZ and WHZ Z-scores, respectively. Overweight and obesity was defined as a WHZ Z-score of greater than +1.04, which corresponds to the 85^th^ percentile.

Demographic characteristics were summarised using frequencies (%) for categorical variables and mean (±SD) for continuous variables. Sex differences in anthropometric and body composition measurements were assessed using the independent t-test while an analysis of covariance (ANCOVA) was used to assess country differences, adjusting for differences in the ages of the infants in the different cohorts. We used a one-tailed one-sample t-test to compare country means to the pooled mean at 6 months for the birth to 6-month cohort, and at 24 months for the 3-to-24-month cohort. Further, we performed cross-sectional analyses to compare body composition between subsamples of children who had an average HAZ (−0.25 > HAZ < +0.25), above-average (≥+0.25) and below-average (≤−0.25) HAZ using an ANCOVA. All continuous outcome variables were assessed for normality and log transformed. Data were back transformed and tabulated as geometric means (±SD). Graphs were generated using the ggplot2 package (version 3.3.5) and presented as means (±SE). All tests were performed at the 5% level of significance using R Studio version 1.1.383 (RStudio, Inc).

## Results

### Description of maternal characteristics

The demographic characteristics of the cohorts are presented in Table 1. Maternal age was youngest in South Africa and India, and oldest in Australia and Brazil. Mothers from India, Sri Lanka, and Pakistan had the lowest pre-pregnancy weight (up to 60 kg) compared to mothers from Brazil, South Africa, and Australia, who weighed about 10 kg more. Years of maternal education were lowest in Sri Lanka, with an average of 11 years of schooling and highest in Brazil, Pakistan, and Australia, with over 14 years of schooling. Exclusive breastfeeding up to 3 months of age was most common in India and Sri Lanka (95.7 & 96.9%), followed by Australia (67.2%), Pakistan (55.1%), Brazil (53.1%), and lowest in South Africa (41.5%). A prevalence of 62.1% of exclusive breastfeeding at 3 months and 28.8% at 6 months was observed in the pooled sample.

**Table 1:** Comparison of maternal factors between country cohorts.

Variables	Australia	Brazil	India	Pakistan	South Africa	Sri Lanka
*N*	*n* = 133	*n* = 293	*n* = 102	*n* = 170	*n*^a^ = 256 & *n*^b^ = 398	*n* = 165
Age (years)	30.5 (5.0)	30.1 (5.7)	26.4 (5.0)	28.4 (4.6)	26.1 (5.7)	29.6 (5.9)
Pre-pregnancy or 1^st^ trimester weight (kg)^c^	68.0 (62.0–80.0)	67.7 (59.8–79.0)	52.0 (47.2–58.0)	60.0 (52.0–67.0)	66.6 (59.8–77.8)	56.0 (48.6–65.2)
Years of education (years)	14.9 (2.5)	14.1 (3.6)	13.3 (2.5)	14.2 (2.7)	12.0 (1.5)	11.5 (1.9)
Marital status *n* (%)						
Single	7 (5.3)	19 (6.5)	0 (0.0)	0 (0.0)	561 (87.1)	1 (0.6)
Married/Cohabiting	126 (94.7)	274 (93.5)	100 (100.0)	170 (100.0)	83 (12.9)	163 (99.4)
Occupation *n* (%)						
Housework	6 (4.5)	41 (14.0)	85 (85.0)	143 (84.1)	108 (22.1)	101 (61.6)
Student	11 (8.3)	15 (5.1)	0 (0.0)	1 (0.6)	64 (13.1)	0 (0.0)
Skilled manual work	90 (67.7)	9 (3.1)	3 (3.0)	23 (13.5)	103 (21.1)	43 (26.2)
Unskilled manual work	2 (1.5)	10 (3.4)	0 (0.0)	2 (1.2)	41 (8.4)	1 (0.6)
Managerial/professional/technical	6 (4.5)	117 (39.9)	10 (10.0)	1 (0.6)	27 (5.5)	14 (8.5)
Clerical support, service or sales	3 (2.3)	97 (33.1)	2 (2.0)	0 (0.0)	17 (3.5)	5 (3.0)
Other	15 (11.3)	4 (1.4)	0 (0.0)	0 (0.0)	129 (26.4)	0 (0.0)
Parity *n* (%)						
None	57 (42.9)	166 (56.5)	57 (57.0)	67 (39.4)	262 (42.7)	79 (48.2)
One	52 (39.1)	93 (31.6)	37 (37.0)	57 (33.5)	206 (33.6)	44 (26.8)
Two+	24 (18.1)	35 (11.9)	6 (6.0)	46 (27.1)	145 (23.7)	41(25.0)
Gestational age (weeks)	39.6 (1.1)	38.9 (0.9)	38.9 (0.9)	39.1 (1.1)	39.1 (1.3)	39.2 (1.2)
Mode of delivery *n* (%)						
Vaginal spontaneous	75 (56.4)	51 (17.4)	9 (9.0)	100 (58.1)	641 (99.5)	96 (58.5)
Vaginal assisted (e.g., forceps, vacuum)	19 (14.3)	1 (0.3)	62 (62.0)	7 (4.7)	3 (0.5)	5 (3.1)
Cesarean section	39 (29.3)	241 (82.3)	29 (29.0)	63 (37.2)	0 (0.0)	62 (37.8)
Assisted breech/ Breech extraction	0 (0.0)	0 (0.0)	0 (0.0)	0 (0.0)	0 (0.0)	1 (0.6)
Exclusive breastfeeding at 3 months	88 (67.2)	112 (53.3)	88 (95.7)	49 (55.1)	117 (41.5)	126 (96.9)

Results presented as mean (±SD), unless otherwise stated.

^a^Sample size for cohort 2 (0–6 months) in South Africa.

^b^Sample size for cohort 1 (3–24 months) in South Africa.

^c^Median (Q1–Q3).

### Maternal and growth characteristics of infants who had vs. no ADP data at 6-mo

Differences in maternal and infant characteristics between participants with ADP and those without are presented in Supplementary Table 1. We could not collect ADP at 6 months on 98 (~30%) participants who were above the weight allowable by the technique or those who could not be pacified. In both sexes, mothers of infants who had no ADP were significantly heavier than those of infants with ADP. Both male and female infants with no ADP were heavier at birth and at 6 months of age than those with ADP. A total 32 (60%) male and 19 (42%) female infants had a weight greater than 8 kg at age 6 months.

### Growth parameters and the prevalence of stunting, wasting, underweight, and overweight

Sex and country differences in weight and length are presented in Supplementary Table 2. In the birth-to-6-month cohort, Australian infants were heavier at birth than both Indian and South African infants. In the 3-to-24-month cohort, Brazilian infants were generally heavier and taller than Pakistani, South African, and Sri Lankan infants at different timepoints.

**Fig. 1:**
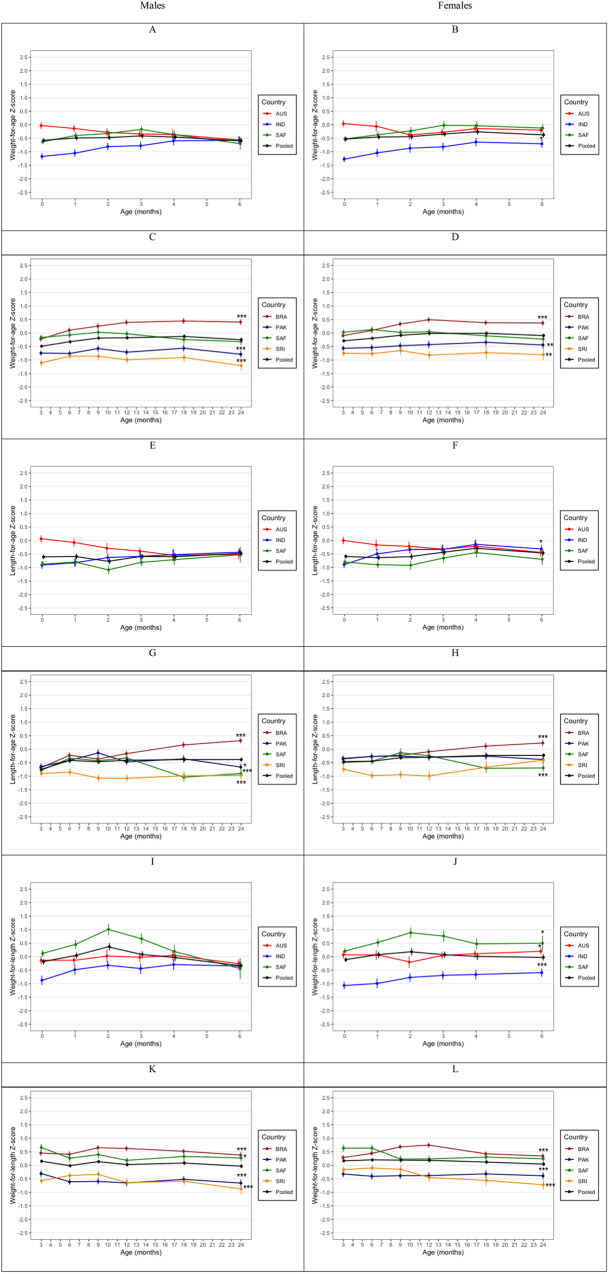
Anthropomtric characteristics of the cohort. Mean (±SE) weight-for-age (**A**–**D**), length-for-age (**E**–**H**), and weight-for-length (**I**–**L**) Z-scores for boys and girls in the birth-to-6-month and 3-to-24-month cohorts. Differences between pooled and country means at 6 and 24 months are denoted with asterisks (*p < 0.05, **p < 0.01, ***p < 0.001).

The mean weight-for-age (WAZ), length-for-age (HAZ), weight-for-length (WHZ) *z* scores by sex are presented in Fig. 1. In both the birth to 6-month and the 3-to-24-month cohorts, the mean WAZ and HAZ for the pooled sample was consistently between −0.5 SDS and the median of child growth standards, although the mean HAZ started below −0.5 SDS for length at and before 3 months. WHZ was consistently close to the median in both cohorts. In the birth to 6-month cohort significant differences were observed in WAZ and HAZ at birth, which reduced as the children grew, disappearing at 6 months. In contrast, the difference in WAZ and HAZ widened as children grew in the 3-to-24-month cohort.

**Fig. 2:**
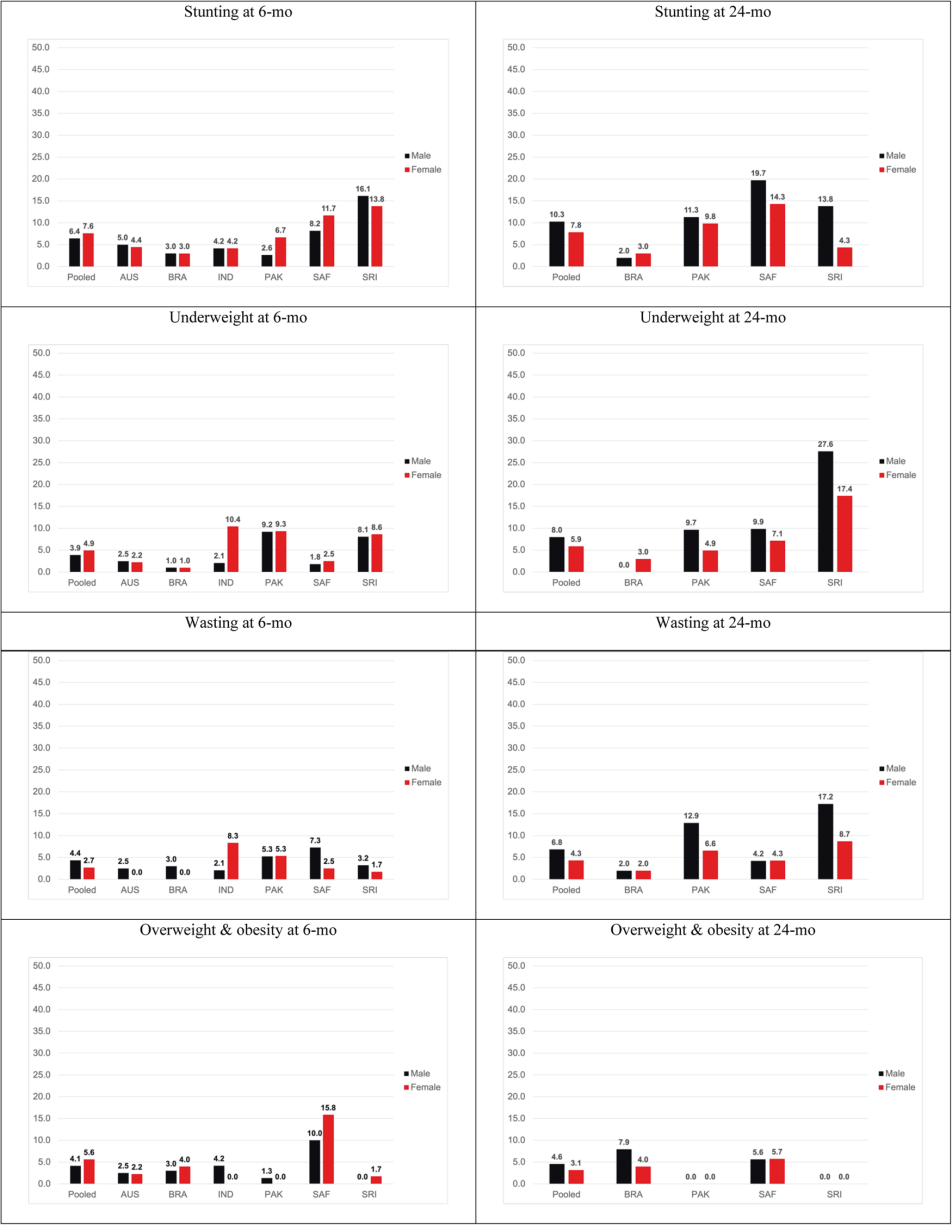
Prevalence (%) of stunting, wasting, and underweight at 6 and 24 months using WHO growth charts.

In the pooled data, boys had a marginally higher prevalence of stunting, underweight, wasting, and overweight and obesity than girls, peaking at 10.3 vs. 7.8%, 8.0 vs. 5.9%, 6.8 vs. 4.3%, and 4.6 vs. 3.1% respectively at 24 months (Fig. 2).

### Sex and country differences in body composition

**Fig. 3:**
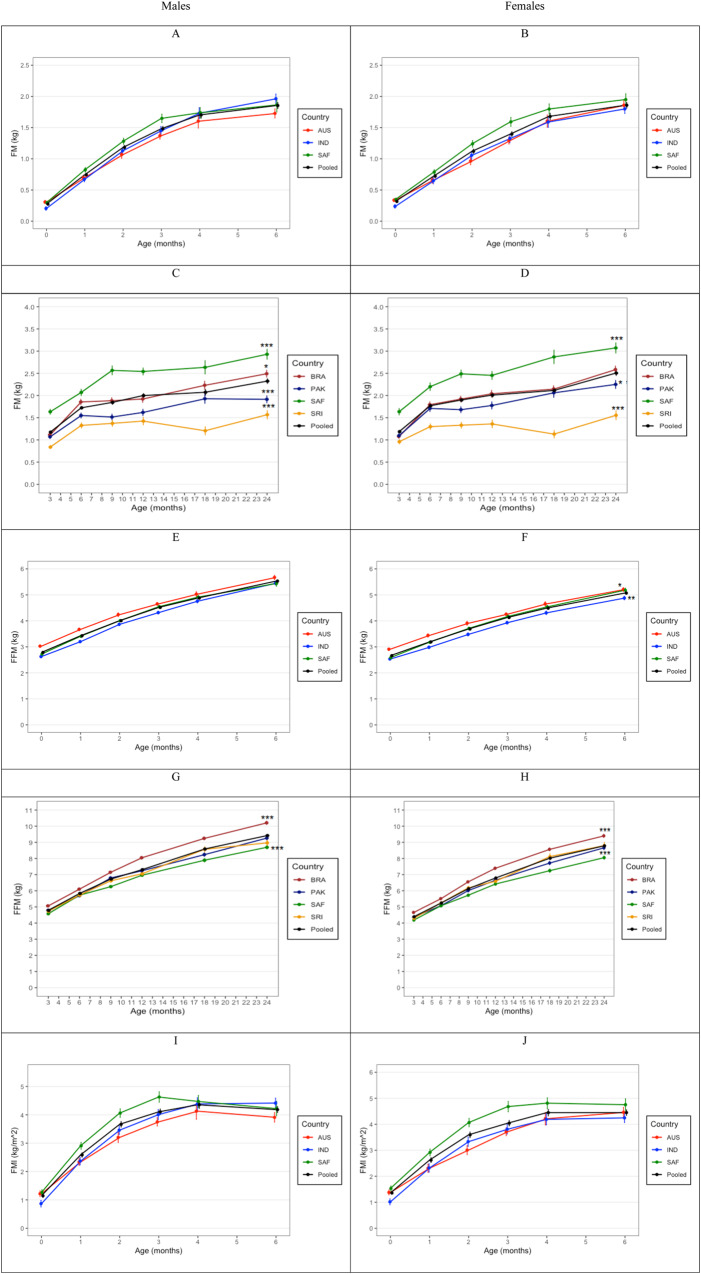

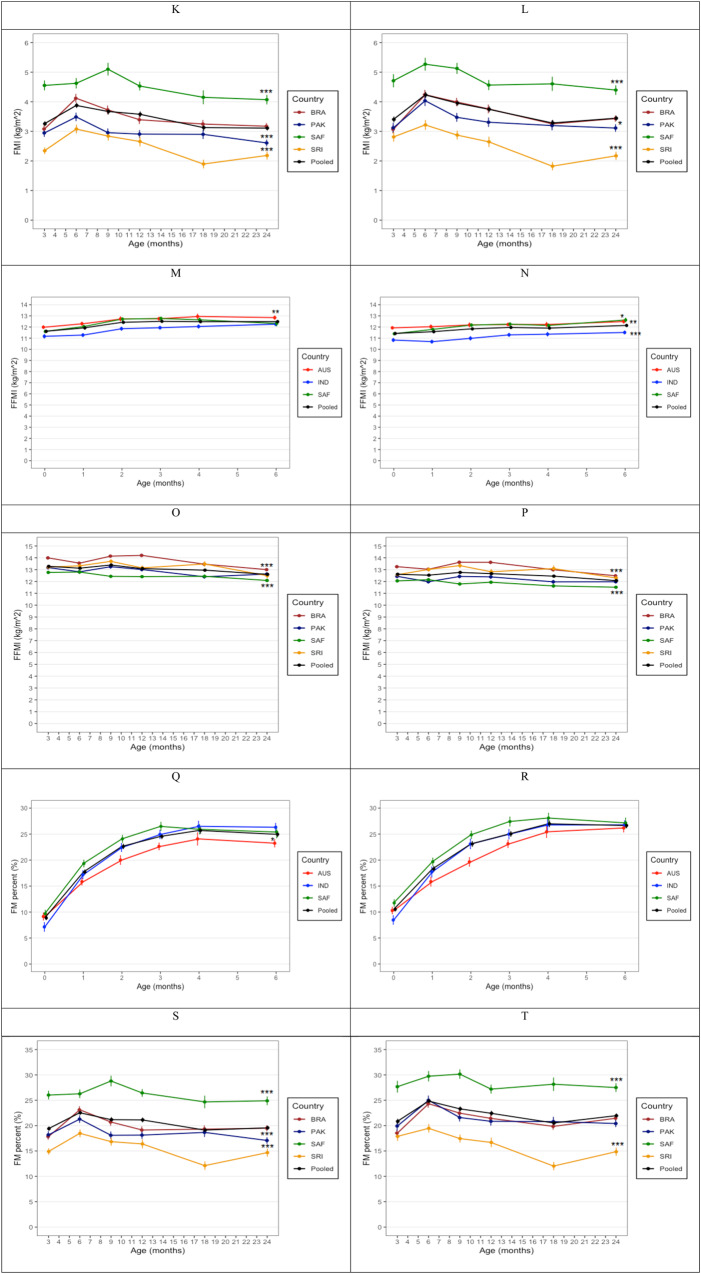
Longitudinal changes in body composition. Mean (±SE) fat mass (**A**–**D**), fat-free mass (**E**–**H**), fat mass index (**I**–**L**), fat-free mass index (**M**–**P**), and fat mass % (**Q**–**T**) for boys and girls in the birth-to-6-month and 3-to-24-month cohorts. Differences between pooled and country means at 6 months and 24 months are denoted with asterisks (*p < 0.05, **p < 0.01, ***p < 0.001).

Differences in FM, FFM, FMI, FFMI, and %FM are presented in Fig. 3 and Supplementary Table 3. The pooled data showed negligible sex differences in FM and FMI in both the birth-to-6-month and 3-to-24-month cohorts. Girls had greater a %FM at birth and 6 months in the birth-to-6-month cohort, and at all ages in the 3-to-24-month cohort. Boys had greater FFM and FFMI at all ages in both cohorts.

In the birth-to-6-month cohort, South African boys and girls had greater FM, FMI and %FM than Australian and Indian infants between birth and 3 months of age. In boys, Australia infants had greater FFM than South African and Indian infants up to 3 months. In girls, Australian and South African infants had greater FFM than Indian infants at all ages. Similar trends were observed in FFMI in both sexes.

In the 3-to-24-month cohort, FM, FMI, and %FM was highest in South African infants and lowest in Sri Lankan infants. Highest FFM and FFMI were found in girls and boys from Brazil, followed by Sri Lanka and Pakistan, with the lowest values observed in South Africa.

### Differences between the pooled and country means at 6 and 24 months

In the birth-to-6-month cohort, there were no differences between country and the pooled means for WAZ, HAZ and WHZ in boys. In girls, Indian infants had lower WAZ (*p* < 0.05) and WHZ (*p* < 0.001) than the pooled mean at 6 months. In contrast, Australian and South African girls had higher WHZ (*p* < 0.05) compared to the pooled mean at 6 months. Australian boys had higher FFMI than the pooled mean at 6 months (*p* < 0.01), while their %FM was lower (*p* < 0.01). Similarly, mean FFM (*p* < 0.01) and FFMI (*p* < 0.001) were lower among Indian girls than the pooled mean at 6 months, but were greater among Australian girls. South African girls also had greater FFMI compared to the pooled mean at 6 months.

In the 3-to-24-month cohort, Brazilian boys and girls had higher WAZ, HAZ, WHZ (*p* < 0.001) than the pooled mean at 24 months. South African boys (*p* < 0.05) also had higher WHZ than the pooled mean at 24 months. WAZ and WHZ was lower among Pakistani and Sri Lankan boys and girls. Pakistani, South African and Sri Lankan boys had lower HAZ than the pooled mean at 24 months. Brazilian boys had greater FM (*p* < 0.05), while both sexes had greater FFM and FFMI (*p* < 0.001) than the pooled mean at 24 months. South African boys and girls had higher FM, FMI, and %FM (*p* < 0.001) but lower FFM and FFMI (*p* < 0.001) than the pooled mean at 24 months. Both Pakistani and Sri Lankan boys and girls had lower FM, FMI and %FM than the pooled mean at 24 months.

### Differences in body composition between sub-samples of HAZ

**Fig. 4:**
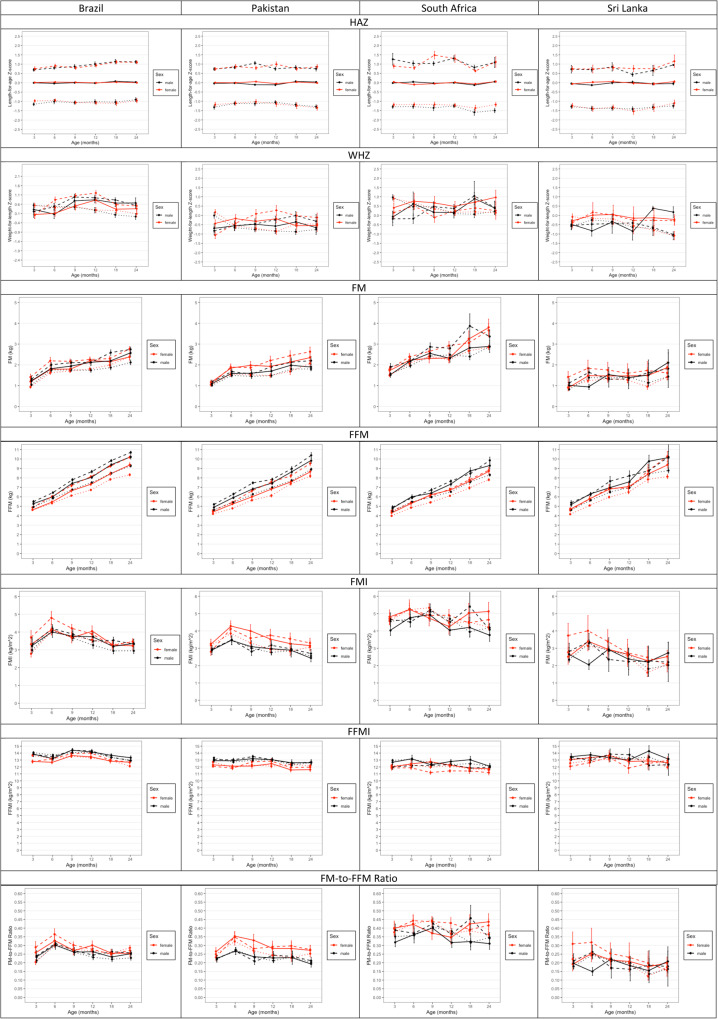
Comparison between infants with average (>−0.25 to <+0.25; solid), above-average (≥+0.25; dashed) and below-average (≤−0.25; dotted) HAZ for height-for-age Z-score, weight-for-length Z-score, fat mass, fat-free mass, fat mass index, fat-free mass index, and fat mass to fat-free mass ratio in the 3-to-24-month cohort. Mean (±SE) are presented.

Differences in HAZ, WHZ, FM, FFM, FMI, FFMI, and FM-to-FFM ratio between infants with average (−0.25 > & < +0.25), below-average (≤ −0.25), and above-average (≥ +0.25) HAZ are presented in Fig. 4. were no significant differences in WHZ except in Brazil and Sri Lanka where boys with below-average HAZ had lower WHZ than those with above-average and average HAZ, respectively. Boys from Brazil (*p* < 0.001) and girls from Pakistan (*p* < 0.05) and South Africa (*p* < 0.05) with below-average HAZ, had lower FM than above-average infants, and those with average HAZ in South Africa (*p* < 0.05). Above-average HAZ girls in Brazil and Pakistan had higher FFM than both average and below-average HAZ girls. Additionally, below-average HAZ boys and girls in Brazil and South Africa, boys in Pakistan and girls in Sri Lanka had lower FFM than both average and above-average HAZ infants. Below-average HAZ girls from Brazil also had lower FFMI than above-average HAZ girls. There were no significant differences in FMI and the FM-to-FFM ratio.

## Discussion

Our aim was to characterise infant body composition longitudinally and to assess how body composition behaves in relation to ideal growth. Women in these cohorts were of above-average socio-economic status, with low parity, and committed to exclusive breastfeeding to provide an optimum environment for child growth. We compared maternal and growth characteristics between infants who had ADP data at 6 months and those who didn’t and found differences in maternal and infant anthropometry. Infants of both sexes for whom ADP data was not collected at 6 months were born to mothers who were heavier before or during early pregnancy, and were consequently heavier at birth and at 6 months. Thus, body composition data in the birth to 6-month cohort represent a lighter population at 6 months, when using ADP. Data for the 3-to-24-month cohort (TBW-derived) may provide a better population estimate for body composition at 6 months irrespective of the method give the strong agreement between ADP-derived and TBW-derived body composition (intraclass correlation of 0.91 for FM and 0.92 for FFM), shown by Kuriyan et al. [8] on the same dataset.

Given the maternal commitment, the prevalence of breastfeeding at 3 months in the pooled sample (62.1%) was higher than the global average (42%) in LMICs [17] and notably higher than reported on average for each country [18, 19]. Consequently, the aggregate growth in height, weight and WHZ aligned with WHO child growth standards for these earlier infant months, except for Sri Lanka, which started about one standard deviation below. Consequently, the prevalence of stunting in this study was lower than regional/country averages. Although the aggregate HAZ maintained a steady level throughout infancy, a decline in South Africa and Pakistan and a lower start in Sri Lanka could have contributed to increase in the prevalence of stunting which peaked by 2 years. We speculate that as the infants age, greater exposure to living conditions, post-weaning practices and infections impact linear growth.

Children from South and Southeast Asia were lighter and had lower FM than infants from Australia, Brazil, and South Africa, and children from South Africa had the highest FM. As anticipated across all countries, infant FM and FFM accrued with age corresponding to increases in linear growth (size). After adjusting for length, for all countries FMI peaked early around 3–6 months and then steadily decreased. Similar patterns were observed across boys and girls, with little ratio differences (FM/FFM). The patterns observed were similar to those observed in other longitudinal studies [11, 20]. However, the pattern for FFMI was different where the peak occurred around 12 months and then slowly decreased. Boys consistently had greater FFM for size than females. While South African infants had greater FMI throughout infancy compared to Brazil, Pakistan and Sri Lanka, the opposite was true for FFMI where it was the lowest. The implication is that the ratio of FMI to FFMI was highest in South Africa, predisposing South African children to greater adiposity and non-communicable disease (NCD) risk trajectories [21].

Santos et al. showed that an increase in economic inequality as measured by the Gini index was associated with a rise in FM and a decrease in FFM in the MIBCRS [9]. Surprisingly, birth weight and exclusive breastfeeding were associated with measures of lean mass as opposed to adiposity at 6 months. However, they showed that continued breastfeeding at 12 months was associated with reduction in FM but also FFM and FFMI, while meeting minimum dietary diversity was associated with positive increase in FFM and FFMI. These findings by Santos et al. [9] on the same cohort for the current study further highlight the strong ecological drivers of adiposity underpinning the differences observed in the current study.

When assessed in relation to ideal linear growth, FMI, FFMI and FM-to-FFM ratio patterns were similar and with less differences between length groups, possibly due to the relatively wide standard errors. There were more differences in FFM across the length groups within each country, but less so between countries and with relatively smaller standard errors. While the overall pattern for FM was similar, FM varied more across the length groups in Brazil and South Africa, with South African females exhibiting greatest FM at 24 months. For all the adiposity indices, South African girls had greater adiposity. This highlights important regional differences for obesity risk that starts in early life even with infants who maintained height close to the median of the WHO growth standard, further emphasising the need to integrate body composition monitoring into paediatric and public health practices to offset childhood obesity.

This study had several strengths and limitations. It is the largest harmonised multi-country infant body composition study completed to date. A limitation of this study was that challenges in administering the deuterium dilution solution to infants resulted in some participants having incomplete longitudinal measurements over time. However, we did factor this in and oversampled to partly compensate for missing data. Secondly, the differences in the assumptions and algorithms of the two methods used to measure body composition separately from birth to 6 months and from 3 to 24 months, do not allow for a smooth transition for longitudinal assessment from birth to 24 months. Lastly, we did not present data on living conditions, post-weaning practices and infections, which can affect linear growth and body composition.

In conclusion, patterns of body composition changes from birth to 2 years of age are similar across the countries and sexes, but the absolute FM and FFM adjusted for length is heterogeneous. This variance may indicate that independent of growth-restricting factors, infant FM and FFM gain are influenced by ethnic and dietary-pattern differences, such that in some settings may be contributing to early childhood obesity. Identifying factors that contribute to these body composition differences is critical. Notwithstanding, measuring body composition remains a research tool until more precise, accurate and quick measurements are achieved, and their clinical applicability established. Future research will need to support ease and accessibility of infant body composition assessments, better understanding the consequences of infant adiposity subgroups for later health and developing efficacious interventions to address early signs of childhood obesity.

## Supplementary information

Consortium members

Supplementary Table 1

Supplementary Table 2

Supplementary Table 3

## Data Availability

Data described in the manuscript, code book, and analytic code will be made publicly and freely available without restriction at: https://witscloud-my.sharepoint.com/personal/00000085_wits_ac_za/_layouts/15/onedrive.aspx?id=%2Fpersonal%2F00000085%5Fwits%5Fac%5Fza%2FDocuments%2FIAEA%20MIBCRS%20Data&ga=1.
