# Supplementary material for: Infant growth and body composition from birth to 24 months: are infants developing the same?
Source: Eur J Clin Nutr. 2024 Jan 3;78(11):952–62. doi: 10.1038/s41430-023-01386-5 (PMC11537950; doi:10.1038/s41430-023-01386-5)
Supplement: Supplementary file 1 — Consortium members [file 41430_2023_1386_MOESM1_ESM.docx]

**Full Consortium Members**

***MIBCRS**: Shabina Ariff^8^, Andrew P Hills^9^, Rebecca Kuriyan^7^, Shane Norris^1,2^, Ina S Santos^6^, V. Pujitha Wickranasinghe^5^, Alexia J Murphy-Alford^4^, Lukhanyo H Nyati^1,3^, Caroline S Costa^6^, Nishani Lucas^5^, Anura V Kurpad^7^, Leila C Ismail^10,11^, Sisitha Jayasinghe^9^. Tanvir Ahmad^10,12^, Kiran DK Ahuja^9^, Jeff M Beckett^9^, Renata M Bielemann^6^, Nuala M Byrne^9^, Laila Charania^8^, Michele P Christian^7^, Priscilla J Divya^7^, Anne Hanley^9^, Manoja P Herath^5^, Pulani Lanerolle^5^, Cornelia Loechl^4^, Najat Moktar^4^, Upul Senerath^5^, Christine Slater^4^, Sajid Soofi^8^, Steven J Street^8^, Neiva CJ Valle^6^, and Ayesha Yameen^12^

^12^Isotope Application Division, Islamabad, Pakistan.
