# Supplementary material for: Infant growth and body composition from birth to 24 months: are infants developing the same?
Source: Eur J Clin Nutr. 2024 Jan 3;78(11):952–62. doi: 10.1038/s41430-023-01386-5 (PMC11537950; doi:10.1038/s41430-023-01386-5)
Supplement: Supplementary file 2 — Supplementary Table 1 [file 41430_2023_1386_MOESM2_ESM.docx]

**Table S1: Comparison of maternal and birth characteristics between participants with vs. without ADP data at 6 months within each infant sex. Data are summarised as mean (±SD), unless otherwise stated.**

| **Characteristics** | **Male** | | **Female** | |
| --- | --- | --- | --- | --- |
|  | **No ADP** | **With ADP** | **No ADP** | **With ADP** |
| N | 53 | 104 | 45 | 119 |
| Age (years) | 29.1 (6.4) | 27.5 (5.3) | 28.2 (6.1) | 27.8 (5.7) |
| Pre-pregnancy or 1st trimester weight (kg)^∂^ | 68.5  (61.8 – 76.5)*** | 59.4  (50.0 – 67.2) | 76.5  (65.0 – 91.2)*** | 59.0  (50.0 – 68.0) |
| Years of education (years)^∂^ | 12 (12 – 15) | 13 (12 – 15) | 12 (12 – 15) | 12 (12 – 15) |
| Marital status n (%) |  |  |  |  |
| Single | 16 (36,1) | 18 (17,3) | 14 (37.5) | 29 (24.8) |
| Married/Cohabiting | 36 (63,9) | 86 (82,7) | 30 (62.5) | 88 (75.2) |
| Mode of delivery n (%) |  |  |  |  |
| Vaginal spontaneous | 44 (83,9) | 81 (77,9) | 37 (84,0) | 91 (78,4) |
| Cesarean section | 9 (16,1) | 23 (22,1) | 8 (16,0) | 25 (21,6) |
| Gestational age (weeks) | 38.9 (1.2)* | 39.3 (1.1) | 39.1 (1.1) | 39.3 (1.2) |
| Child weight at birth (kg) | 3.3 (0.5)** | 3.1 (0.6) | 3.2 (0.6)** | 2.9 (0.6) |
| Child length at birth (cm) | 49.5 (2.3) | 49.1 (2.7) | 48.4 (2.6) | 48.0 (2.3) |
| Child weight at 6 mo (kg) | 8.4 (1.5)*** | 7.4 (1.0) | 7.7 (1.4)*** | 7.0 (0.9) |
| Child length at 6 mo (cm) | 67.5 (3.0) | 66.6 (2.8) | 65.6 (4.1)* | 64.8 (2.4) |

*p<0.05; **p<0.01; ***p<0.001

^∂^Median (Q1 – Q3)
