# Supplementary material for: Infant growth and body composition from birth to 24 months: are infants developing the same?
Source: Eur J Clin Nutr. 2024 Jan 3;78(11):952–62. doi: 10.1038/s41430-023-01386-5 (PMC11537950; doi:10.1038/s41430-023-01386-5)
Supplement: Supplementary file 3 — Supplementary Table 2 [file 41430_2023_1386_MOESM3_ESM.docx]

**Table S2: Sex and country comparisons in (i) weight and (ii) length in the 0 to 6-mo and 3-to-24-mo cohorts. Sex differences are denoted with asterisks (*p<0.05, **p<0.01, ***p<0.001) and country differences with a letter (a to f)^†^ and digit (^1^p<0.05, ^2^p<0.01, ^3^p<0.001) combination.**

1. **Birth-to-6-mo cohort**

| Variable | Australia | | India | | | South Africa | | | | Pooled | | | |
| --- | --- | --- | --- | --- | --- | --- | --- | --- | --- | --- | --- | --- | --- |
|  | Male | Female | Male | Female | | Male | | Female | Male | | | | Female |
| i) Weight (kg) |  | | | | | | | | | | | | |
| Birth | 3.4 (0.4)^a3,b3^  n=65 | 3.3 (0.4)^a3,b3^  n=63 | 2.9 (0.4)^*^  n=51 | | 2.8 (0.4)  n=49 | | 3.1 (0.4)  n=104 | 3.0 (0.4)  n=111 | | | 3.1 (0.4)^*^  n=220 | 3 (0.4)  n=224 | |
| 1 mo | 4.4 (0.5)^a3^  n=44 | 4.2 (0.6)^a3^  n=36 | 3.9 (0.5)^**;c2^  n=28 | | 3.7 (0.5)^c2^  n=36 | | 4.3 (0.5)^*^  n=67 | 4.0 (0.6)  n=85 | | | 4.2 (0.5)^***^  n=149 | 4 (0.6)  n=157 | |
| 2 mo | 5.3 (0.5)^**^  n=27 | 4.9 (0.7)  n=32 | 5 (0.5)^***;c1^  n=43 | | 4.6 (0.7)^c1^  n=40 | | 5.3 (0.5)^**^  n=60 | 5.0 (0.7)  n=71 | | | 5.2 (0.5)^***^  n=130 | 4.8 (0.7)  n=143 | |
| 3 mo | 6.1 (0.6)^***^  n=55 | 5.6 (0.7)  n=63 | 5.8 (0.6)^***;c2^  n=44 | | 5.3 (0.7)^c2^  n=48 | | 6.2 (0.6)^**^  n=50 | 5.8 (0.7)  n=56 | | | 6.0 (0.6)^***^  n=149 | 5.6 (0.7)  n=167 | |
| 4 mo | 6.7 (0.7)  n=22 | 6.3 (0.8)  n=29 | 6.5 (0.7)^***^  n=37 | | 5.9 (0.7)^c1^  n=38 | | 6.7 (0.7)  n=36 | 6.4 (0.8)  n=49 | | | 6.6 (0.7)^***^  n=95 | 6.2 (0.7)  n=116 | |
| 6-mo | 7.4 (0.8)^*^  n=40 | 7.1 (0.8)  n=45 | 7.5 (0.8)^***^  n=48 | | 6.7 (0.7)^c1^  n=48 | | 7.3 (0.7)  n=18 | 7.2 (0.7)  n=32 | | | 7.4 (0.7)^***^  n=106 | 7.0 (0.7)  n=125 | |
| ii) Length (cm) |  | | | | | | | | | | | | |
| Birth | 50.3 (1.8)^**;a3,b3^ | 49.4 (1.7)^a1,b3^ | 48.5 (2.2) | 48.4 (2.1) | | 48.4 (1.9)^*^ | | 47.6 (1.8) | 49 (1.9)^***^ | | | | 48.3 (1.8) |
| 1 mo | 54.7 (2.0)^*;a2,b3^ | 53.5 (2.2)^b2^ | 53.3 (1.9) | 52.9 (2.1) | | 53.2 (1.9)^**^ | | 51.9 (2.1) | 53.6 (2.1)^***^ | | | | 52.5 (2.2) |
| 2 mo | 57.7 (2.0)^b2^ | 56.6 (2.3)^b1^ | 57.1 (2)^c1^ | 56.3 (2.4) | | 56.2 (2.0)^*^ | | 55.2 (2.3) | 56.8 (2.1)^***^ | | | | 55.8 (2.5) |
| 3 mo | 60.4 (2.0)^***^ | 59 (2.4) | 60.2 (2)^*^ | 59 (2.4) | | 59.7 (1.9)^**^ | | 58.3 (2.3) | 60.1 (2.1)^***^ | | | | 58.8 (2.4) |
| 4 mo | 62.3 (2.4) | 61.7 (2.3) | 62.8 (2.3)^*^ | 61.6 (2.3) | | 62.3 (2.2)^*^ | | 61.1 (2.3) | 62.5 (2.2)^***^ | | | | 61.4 (2.4) |
| 6-mo | 66.5 (2.2)^***^ | 64.6 (2.3) | 66.7 (2.2)^***^ | 65.1 (2.4) | | 66.4 (2.1)^**^ | | 64 (2.2) | 66.6 (2.1)^***^ | | | | 64.6 (2.3) |

^†^a=Australia vs India; b=Australia vs South Africa; c=India vs South Africa

*p<0.05, **p<0.01, ***p<0.001

^1^p<0.05, ^2^p<0.01, ^3^p<0.001

1. **3-to-24-mo cohort**

| Variable | Brazil | | Pakistan | | South Africa | | Sri Lanka | | | | Pooled | | | | |
| --- | --- | --- | --- | --- | --- | --- | --- | --- | --- | --- | --- | --- | --- | --- | --- |
|  | Male | Female | Male | Female | Male | Female | Male | | Female | | Male | | Female | |  |
| i) Weight (kg) |  | | | | | | | | | | | | |  |  |
| 3 mo | 6.2 (0.7)^***;a2,c3^  n=104 | 5.8 (0.6)^a2,c3^  n=108 | 5.9 (0.7)^***;d2^  n=79 | 5.5 (0.6)^d3^  n=71 | 6.3 (0.8)^**;f3^  n=114 | 5.9 (0.7)^f3^  n=92 | 5.6 (0.7)^**^  n=74 | | 5.4 (0.6)  n=66 | | 6.1 (0.8)^***^  n=371 | | 5.7 (0.7)  n=337 | |  |
| 6-mo | 8.0 (0.9)^***;a3,c3^  n=100 | 7.4 (0.9)^a3,c3^  n=101 | 7.3 (0.8)^***;d3^  n=76 | 6.8 (0.8)^d3^  n=75 | 7.9 (0.9)^**;f3^  n=92 | 7.4 (0.9)^f3^  n=88 | 7.2 (0.8)^***^  n=62 | | 6.7 (0.8)  n=58 | | 7.6 (0.9)^***^  n=330 | | 7.1 (0.9)  n=322 | |  |
| 9 mo | 9.1 (1)^***;a3,c3^  n=101 | 8.5 (1.1)^a3,c3^  n=109 | 8.4 (0.9)^***;d2^  n=64 | 7.8 (1)^d1^  n=70 | 8.9 (1.0)^***;f3^  n=82 | 8.2 (1.1)^f2^  n=94 | 8.1 (0.9)^**^  n=56 | | 7.6 (1.0)  n=51 | | 8.7 (1)^***^  n=303 | | 8.1 (1.1)  n=324 | |  |
| 12 mo | 10.0 (1.2)^**;a3,b1,c3^  n=86 | 9.5 (1.3)^a2,b1,c3^  n=97 | 8.9 (1)^*;d3^  n=64 | 8.5 (1.1)^d1^  n=65 | 9.6 (1.1)^***;f3^  n=129 | 9.0 (1.2)^f3^  n=103 | 8.7 (1.0)^**^  n=52 | | 8.1 (1.1)  n=36 | | 9.5 (1.1)^***^  n=321 | | 9 (1.3)  n=301 | |  |
| 18 mo | 11.6 (1.4)^***;a3,b3,c3^  n=94 | 10.8 (1.3)^a3,b1,c3^  n=105 | 10.3 (1.2)^*^  n=57 | 9.9 (1.2)  n=56 | 10.7 (1.3)  n=54 | 10.2 (1.3)^f1^  n=51 | 10.0 (1.2)  n=29 | | 9.4 (1.2)  n=25 | | 10.8 (1.4)^***^  n=234 | | 10.3 (1.4)  n=237 | |  |
| 24-mo | 12.8 (1.6)^**;a3,b3,c3^  n=101 | 12.1 (1.6)^a3,b2,c3^  n=101 | 11.2 (1.4)  n=62 | 11.0 (1.5)  n=61 | 11.8 (1.4)^*;f2^  n=71 | 11.2 (1.5)  n=70 | 10.7 (1.3)  n=29 | | 10.5 (1.4)  n=23 | | 11.9 (1.6)^***^  n=263 | | 11.4 (1.6)  n=255 | |  |
| ii) Length (cm) |  | | | | | | | | | | | | |  |  |
| 3 mo | 60.2  (2.1)^*^ | 59.3  (2.1)^c1^ | 60.3  (2.2)^**^ | 59.3  (2.1) | 59.9 (2.2)^**^ | 58.9 (2.1) | 59.8 (2.2)^***^ | 58.4 (2.1) | | 60 (2.2)^***^ | | 59 (2.2) | | | |
| 6-mo | 67.1  (2.4)^***;c2^ | 65.0  (2.3)^c3^ | 66.7  (2.4)^***^ | 65.1  (2.3)^e3^ | 66.9 (2.4)^***;f1^ | 64.6 (2.3)^f1^ | 65.6 (2.4)^***^ | 63.5 (2.3) | | 66.7 (2.4)^***^ | | 64.7 (2.3) | | | |
| 9 mo | 71.1  (2.5)^***;c2^ | 69.3  (2.9) | 71.6  (2.5)^***;e3^ | 69.6  (2.9)^e1^ | 70.9 (2.4)^**;f2^ | 69.7 (2.9)^f2^ | 69.5 (2.5)^***^ | 68 (2.9) | | 70.9 (2.6)^***^ | | 69.3 (2.9) | | | |
| 12 mo | 75.3  (2.8)^***;a3,c2^ | 73.7  (3.1)^c2^ | 74.6  (2.7)^**^ | 73.3  (3.0) | 74.9 (2.7)^***;f2^ | 73.3 (3.0)^f1^ | 73.2 (2.8)^**^ | 71.7 (3.1) | | 74.7 (2.7)^***^ | | 73.2 (3.1) | | | |
| 18 mo | 82.9 (3.1)^**;a1,b3,c3^ | 81.2  (3.4)^b3,c2^ | 81.5  (3.1^)*;d2^ | 80.3  (3.4) | 79.7 (3.0) | 78.9 (3.3) | 79.7 (3.1) | 78.7 (3.4) | | 81.4 (3.4)^***^ | | 80.2 (3.6) | | | |
| 24-mo | 88.6 (3.4)^***;a3,b3,c3^ | 86.8 (3.5)^a1,b3,c1^ | 85.7  (3.4) | 85.0  (3.5) | 84.8 (3.3) | 83.6 (3.4) | 84.7 (3.3) | 84.6 (3.4) | | 86.4 (3.7)^***^ | | 85.3 (3.7) | | | |

**^†^**a=Brazil vs Pakistan; b=Brazil vs South Africa; c=Brazil vs Sri Lanka; d=Pakistan vs South Africa; e=Pakistan vs Sri Lanka, f=South Africa vs Sri Lanka

*p<0.05, **p<0.01, ***p<0.001

^1^p<0.05, ^2^p<0.01, ^3^p<0.001
