# Supplementary material for: Infant growth and body composition from birth to 24 months: are infants developing the same?
Source: Eur J Clin Nutr. 2024 Jan 3;78(11):952–62. doi: 10.1038/s41430-023-01386-5 (PMC11537950; doi:10.1038/s41430-023-01386-5)
Supplement: Supplementary file 4 — Supplementary Table 3 [file 41430_2023_1386_MOESM4_ESM.docx]

**Table S3: Country and sex comparisons in (a) ADP derived (birth to 6-mo) and (b) TBW derived (3 to 24-mo) (i) FM, (ii) FFM, (iii) FMI, (iv) FFMI, and (v) FM%. Sex differences are denoted with asterisks (*p<0.05, **p<0.01, ***p<0.001) and country differences with a letter (a to f)^†^ and digit (^1^p<0.05, ^2^p<0.01, ^3^p<0.001) combination.**

1. **Birth-to-6-mo (air-displacement plethysmography-derived)**

| Variable | Australia | | India | | South Africa | | Pooled | | |
| --- | --- | --- | --- | --- | --- | --- | --- | --- | --- |
|  | Male | Female | Male | Female | Male | Female | Male | | Female |
| i) FM (kg) |  | | | | | | | | |
| Birth | 0.3 (0.2)^a2^ | 0.3 (0.2)^a2^ | 0.2 (0.2)^c1^ | 0.2 (0.2)^c2^ | 0.3 (0.3)^**^ | 0.3 (0.2) | | 0.3 (0.2)^**^ | 0.3 (0.2) |
| 1 mo | 0.7 (0.2)^b1^ | 0.7 (0.3) | 0.7 (0.2)^c1^ | 0.6 (0.3)^c1^ | 0.8 (0.3) | 0.8 (0.4) | | 0.7 (0.3) | 0.7 (0.3) |
| 2 mo | 1.1 (0.3)^b1^ | 1 (0.3)^b2^ | 1.1 (0.3) | 1.1 (0.4) | 1.3 (0.4) | 1.2 (0.4) | | 1.2 (0.4) | 1.1 (0.4) |
| 3 mo | 1.4 (0.4)^b2^ | 1.3 (0.4)^b2^ | 1.5 (0.4) | 1.3 (0.5)^c1^ | 1.6 (0.5) | 1.6 (0.5) | | 1.5 (0.4) | 1.4 (0.5) |
| 4 mo | 1.6 (0.5) | 1.6 (0.5) | 1.7 (0.5) | 1.6 (0.5) | 1.7 (0.5) | 1.8 (0.6) | | 1.7 (0.5) | 1.7 (0.5) |
| 6-mo | 1.7 (0.5) | 1.9 (0.5) | 2 (0.5) | 1.8 (0.5) | 1.9 (0.5) | 1.9 (0.5) | | 1.9 (0.5) | 1.9 (0.5) |
| ii) FFM (kg) |  | | | | | | | | |
| Birth | 3 (0.3)^*;a3,b3^ | 2.9 (0.3)^a3,b3^ | 2.6 (0.3)^***^ | 2.5 (0.3) | 2.7 (0.3)^**^ | 2.6 (0.3) | | 2.8 (0.3)^***^ | 2.7 (0.3) |
| 1 mo | 3.7 (0.4)^*;a3,b3^ | 3.4 (0.4)^a3,b2^ | 3.2 (0.3)^***;c2^ | 3 (0.3)^c1^ | 3.4 (0.3)^***^ | 3.2 (0.4) | | 3.4 (0.4)^***^ | 3.2 (0.4) |
| 2 mo | 4.2 (0.4)^**;a3,b1^ | 3.9 (0.4)^a3^ | 3.9 (0.3)^***^ | 3.5 (0.4)^c1^ | 4 (0.3)^***^ | 3.7 (0.4) | | 4 (0.4)^***^ | 3.7 (0.4) |
| 3 mo | 4.7 (0.4)^***;a3^ | 4.3 (0.4)^a3^ | 4.3 (0.4)^***;c2^ | 3.9 (0.4)^c1^ | 4.5 (0.4)^***^ | 4.2 (0.4) | | 4.5 (0.4)^***^ | 4.1 (0.4) |
| 4 mo | 5 (0.4)^**^ | 4.7 (0.4)^a2^ | 4.8 (0.4)^***^ | 4.3 (0.4)^c1^ | 4.9 (0.4)^***^ | 4.5 (0.4) | | 4.9 (0.4)^***^ | 4.5 (0.4) |
| 6-mo | 5.7 (0.5)^***^ | 5.2 (0.5)^a2^ | 5.4 (0.5)^***^ | 4.9 (0.5)^c1^ | 5.4 (0.5)^*^ | 5.2 (0.5) | | 5.5 (0.5)^***^ | 5.1 (0.5) |
| iii) FMI (kg/m^2) |  | | | | | | | | |
| Birth | 1.2 (0.8)^a1^ | 1.4 (0.8)^a1^ | 0.9 (0.8)^c2^ | 1 (0.8)^c3^ | 1.3 (1)^**^ | 1.5 (1) | | 1.1 (0.8)^***^ | 1.4 (0.8) |
| 1 mo | 2.3 (0.8)^b2^ | 2.3 (1)^b2^ | 2.4 (0.8)^c2^ | 2.3 (1)^c2^ | 2.9 (1) | 2.9 (1.2) | | 2.6 (0.9) | 2.6 (1.2) |
| 2 mo | 3.2 (0.9)^b3^ | 3 (1)^b3^ | 3.5 (1)^c1^ | 3.3 (1.1)^c2^ | 4.1 (1.2) | 4.1 (1.4) | | 3.7 (1.1) | 3.6 (1.3) |
| 3 mo | 3.8 (1.1)^b3^ | 3.7 (1.2)^b3^ | 4 (1.2)^c1^ | 3.8 (1.3)^c2^ | 4.6 (1.3) | 4.7 (1.5) | | 4.1 (1.2) | 4 (1.4) |
| 4 mo | 4.1 (1.3) | 4.2 (1.3) | 4.4 (1.4) | 4.2 (1.3) | 4.5 (1.3) | 4.8 (1.5) | | 4.4 (1.2) | 4.4 (1.4) |
| 6-mo | 3.9 (1.1)^*^ | 4.5 (1.3) | 4.4 (1.2) | 4.2 (1.2) | 4.2 (1.1) | 4.8 (1.3) | | 4.2 (1.1) | 4.4 (1.2) |
| iv) FFMI (kg/m^2) |  | | | | | | | | |
| Birth | 12 (1)^a3,b1^ | 11.9 (0.9)^a3,b3^ | 11.2 (1.2)^*^ | 10.8 (1)^c1^ | 11.6 (1.1) | 11.4 (1) | | 11.6 (1)^*^ | 11.4 (1) |
| 1 mo | 12.3 (0.9)^a3^ | 12 (0.9)^a3^ | 11.3 (0.9)^**;c3^ | 10.7 (0.8)^c3^ | 12 (0.9) | 11.8 (0.9) | | 11.9 (1)^**^ | 11.6 (1.1) |
| 2 mo | 12.7 (1)^**;a3^ | 12.2 (1.1)^a3^ | 11.8 (0.9)^***;c3^ | 11 (1)^c3^ | 12.7 (1)^**^ | 12.2 (1.1) | | 12.4 (1)^***^ | 11.8 (1.1) |
| 3 mo | 12.7 (1)^**;a3^ | 12.2 (1)^a3^ | 11.9 (0.9)^***;c3^ | 11.3 (1)^c3^ | 12.8 (0.9)^*^ | 12.3 (1) | | 12.5 (1)^***^ | 12 (1.1) |
| 4 mo | 12.9 (1)^**;a2^ | 12.2 (1)^a2^ | 12 (0.9)^***;c1^ | 11.4 (1)^c2^ | 12.6 (0.9)^*^ | 12.1 (1) | | 12.5 (0.9)^***^ | 11.9 (1.1) |
| 6-mo | 12.8 (1)^a1^ | 12.5 (1)^a3^ | 12.3 (1)^***^ | 11.5 (0.9)^c3^ | 12.3 (0.9) | 12.6 (1) | | 12.5 (0.9)^**^ | 12.1 (1) |
| v) FM % |  | | | | | | | | |
| Birth | 9.1 (5.4) | 10.3 (5) | 7.1 (5.9)^c1^ | 8.5 (5.8)^c2^ | 9.7 (6.8)^***^ | 11.7 (6.7) | | 8.9 (5.4)^***^ | 10.5 (5.3) |
| 1 mo | 15.9 (4.2)^b3^ | 15.9 (5.2)^b2^ | 17.2 (4.7) | 17.7 (5.8) | 19.3 (5.3) | 19.7 (6.5) | | 17.7 (5.1) | 18.3 (6.3) |
| 2 mo | 20 (4.3)^b3^ | 19.7 (4.7)^a1,b3^ | 22.4 (5) | 23.1 (5.8) | 24.1 (5.2) | 24.9 (6) | | 22.6 (5.1) | 23.1 (5.9) |
| 3 mo | 22.6 (4.9)^b3^ | 23.1 (5.6)^b3^ | 24.9 (5.4) | 25 (6.3) | 26.5 (5.5) | 27.4 (6.5) | | 24.6 (5.4) | 25 (6.2) |
| 4 mo | 24.1 (5.6) | 25.4 (5.9) | 26.5 (5.8) | 26.8 (6.3) | 25.9 (5.4) | 28.1 (6.4) | | 25.7 (5.4) | 27 (6.1) |
| 6-mo | 23.2 (4.5)^**;a1^ | 26.2 (5.3) | 26.3 (5.1) | 26.8 (5.5) | 25.4 (4.7) | 27.2 (5.2) | | 25 (4.8)^**^ | 26.7 (5.1) |

**^†^**a=Australia vs India; b=Australia vs South Africa; c=India vs South Africa

*p<0.05, **p<0.01, ***p<0.001

^1^p<0.05, ^2^p<0.01, ^3^p<0.001

1. **3-to-24-mo (total body water-derived)**

| Variable | Brazil | | Pakistan | | South Africa | | Sri Lanka | | Pooled | |
| --- | --- | --- | --- | --- | --- | --- | --- | --- | --- | --- |
|  | Male | Female | Male | Female | Male | Female | Male | Female | Male | Female |
| i) FM (kg) |  | | | | | | | | | |
| 3 mo | 1.1 (0.5)^b3,c3^ | 1.1 (0.5)^b3^ | 1.1 (0.4)^d3,e3^ | 1.1 (0.5)^d3^ | 1.6 (0.7)^f3^ | 1.6 (0.8)^f3^ | 0.8 (0.4) | 1 (0.4) | 1.2 (0.6) | 1.2 (0.6) |
| 6-mo | 1.9 (0.7)^a2,c3^ | 1.8 (0.7)^b3,c3^ | 1.5 (0.6)^*;d3,e1^ | 1.7 (0.7)^d3,e3^ | 2.1 (0.8)^f3^ | 2.2 (0.9)^f3^ | 1.3 (0.5) | 1.3 (0.5) | 1.7 (0.7) | 1.8 (0.8) |
| 9 mo | 1.9 (0.7)^a3,b3,c3^ | 1.9 (0.7)^b3,c3^ | 1.5 (0.6)^d3^ | 1.7 (0.6)^d3,e2^ | 2.6 (1)^f3^ | 2.5 (0.9)^f3^ | 1.4 (0.5) | 1.3 (0.5) | 1.8 (0.9) | 1.9 (0.8) |
| 12 mo | 1.9 (0.8)^a1,b3,c3^ | 2 (0.8)^b2,c3^ | 1.6 (0.6)^d3^ | 1.8 (0.7)^d3,e2^ | 2.5 (1)^f3^ | 2.5 (1)^f3^ | 1.4 (0.6) | 1.4 (0.6) | 2 (0.9) | 2 (0.9) |
| 18 mo | 2.2 (1)^c3^ | 2.1 (0.8)^b3,c3^ | 1.9 (0.8)^d3,e3^ | 2.1 (0.8)^d3,e3^ | 2.6 (1.2)^f3^ | 2.9 (1.1)^f3^ | 1.2 (0.5) | 1.1 (0.5) | 2.1 (1.1) | 2.1 (1) |
| 24-mo | 2.5 (0.9)^a3,b2,c3^ | 2.6 (0.9)^a1,b2,c3^ | 1.9 (0.7)^***;d3,e1^ | 2.3 (0.8)^d3,e3^ | 2.9 (1)^f3^ | 3.1 (1)^f3^ | 1.6 (0.5) | 1.6 (0.5) | 2.3 (1)^*^ | 2.5 (1) |
| ii) FFM (kg) |  | | | | | | | | | |
| 3 mo | 5.1 (0.6)^***;a2,b3,c2^ | 4.7 (0.5)^a2,b3,c3^ | 4.8 (0.6)^***;d1^ | 4.4 (0.5) | 4.6 (0.5)^***^ | 4.2 (0.5) | 4.7 (0.6)^***^ | 4.3 (0.5) | 4.8 (0.6)^***^ | 4.4 (0.5) |
| 6-mo | 6.1 (0.7)^***;a3,b3,c2^ | 5.5 (0.7)^a3,b3^ | 5.7 (0.6)^***^ | 5.1 (0.6) | 5.7 (0.6)^***^ | 5.1 (0.6) | 5.7 (0.7)^***^ | 5.2 (0.6) | 5.8 (0.7)^***^ | 5.2 (0.7) |
| 9 mo | 7.1 (0.9)^***;a1,b3,c2^ | 6.6 (0.9)^a3,b3^ | 6.8 (0.8)^***;d3^ | 6 (0.8) | 6.3 (0.7)^***;f1^ | 5.7 (0.8)^f2^ | 6.6 (0.8)^**^ | 6.2 (0.9) | 6.7 (0.9)^***^ | 6.1 (0.9) |
| 12 mo | 8 (1)^***;a3,b3,c3^ | 7.4 (1)^a3,b3^ | 7.2 (0.8)^***^ | 6.7 (0.9)^d3^ | 7 (0.8)^***^ | 6.4 (0.8) | 7.1 (0.9)^*^ | 6.6 (0.9) | 7.3 (0.9)^***^ | 6.8 (1) |
| 18 mo | 9.2 (1.1)^***;a3,b3,c1^ | 8.6 (1.1)^a3,b3^ | 8.2 (1.0)^**^ | 7.7 (1.0)^d1^ | 7.9 (0.9)^***;f1^ | 7.2 (0.9)^f2^ | 8.6 (1.0) | 8.1 (1.0) | 8.6 (1.1)^***^ | 8 (1.1) |
| 24-mo | 10.2 (1.3)^***;a3,b3,c3^ | 9.4 (1.2)^a3,b3^ | 9.3 (1.2)^***;d1^ | 8.7 (1.1)^d2^ | 8.7 (1.1)^***^ | 8.1 (1.0)^f1^ | 9 (1.1) | 8.8 (1.1) | 9.4 (1.3)^***^ | 8.8 (1.2) |
| iii) FMI (kg/m^2) |  | | | | | | | | | |
| 3 mo | 3.1 (1.2)^b3,c3^ | 3.1 (1.4)^b3^ | 2.9 (1.1)^d3,e3^ | 3.1 (1.4)^d3^ | 4.6 (1.8)^f3^ | 4.7 (2.1)^f3^ | 2.3 (0.9)^*^ | 2.8 (1.3) | 3.3 (1.6) | 3.4 (1.7) |
| 6-mo | 4.1 (1.5)^a2,c3^ | 4.2 (1.6)^b3,c3^ | 3.5 (1.2)^**;d3^ | 4 (1.5)^d3,e2^ | 4.6 (1.7)^*;f3^ | 5.3 (2)^f3^ | 3.1 (1.1) | 3.2 (1.2) | 3.9 (1.5)^**^ | 4.2 (1.8) |
| 9 mo | 3.7 (1.5)^a3,b3,c3^ | 4 (1.4)^a1,b3,c3^ | 3 (1.1)^**;d3^ | 3.5 (1.2)^d3,e2^ | 5.1 (1.9)^f3^ | 5.1 (1.8)^f3^ | 2.8 (1.1) | 2.9 (1.1) | 3.7 (1.7)^*^ | 4 (1.6) |
| 12 mo | 3.4 (1.3)^*;a1,b3,c2^ | 3.8 (1.4)^b3,c3^ | 2.9 (1.1)^*;d3^ | 3.3 (1.2)^d3,e1^ | 4.5 (1.7)^f3^ | 4.6 (1.7)^f3^ | 2.7 (1) | 2.6 (1) | 3.6 (1.6) | 3.7 (1.6) |
| 18 mo | 3.2 (1.3)^b3,c3^ | 3.3 (1.2)^b3,c3^ | 2.9 (1.2)^d3,e3^ | 3.2 (1.2)^d3,e3^ | 4.2 (1.7)^f3^ | 4.6 (1.7)^f3^ | 1.9 (0.8) | 1.8 (0.7) | 3.1 (1.5) | 3.3 (1.5) |
| 24-mo | 3.2 (1.0)^a2,b3,c3^ | 3.4 (1.0)^b3,c3^ | 2.6 (0.9)^***;d3,e1^ | 3.1 (1.0)^d3,e3^ | 4.1 (1.3)^f3^ | 4.4 (1.3)^f3^ | 2.2 (0.7) | 2.2 (0.7) | 3.1 (1.3)^**^ | 3.4 (1.3) |
| iv) FFMI (kg/m^2) |  | | | | | | | | | |
| 3 mo | 14 (1.4)^***;a2,b3^ | 13.3 (1.4)^a3,b3,c2^ | 13.2 (1.3)^***^ | 12.4 (1.3) | 12.8 (1.3)^***^ | 12.1 (1.3) | 13.2 (1.4)^**^ | 12.6 (1.3) | 13.3 (1.4)^***^ | 12.6 (1.4) |
| 6-mo | 13.5 (1.3)^**;a2,b3^ | 13 (1.3)^a3,b3^ | 12.8 (1.3)^***^ | 12 (1.2)^e3^ | 12.8 (1.3)^*^ | 12.2 (1.2)^f3^ | 13.3 (1.4) | 13 (1.3) | 13.1 (1.3)^***^ | 12.5 (1.3) |
| 9 mo | 14.1 (1.4)^**;a3,b3^ | 13.6 (1.5)^a3,b3^ | 13.2 (1.2)^***;d3^ | 12.4 (1.4)^d1,e2^ | 12.4 (1.1)^**;f3^ | 11.8 (1.3)^f3^ | 13.7 (1.3) | 13.3 (1.5) | 13.4 (1.4)^***^ | 12.8 (1.6) |
| 12 mo | 14.2 (1.3)^***;a3,b3,c3^ | 13.6 (1.4)^a3,b3,c1^ | 13 (1.2)^**;d2^ | 12.4 (1.3) | 12.4 (1.1)^**;f2^ | 11.9 (1.2)^f2^ | 13.1 (1.3) | 12.8 (1.4) | 13.1 (1.4)^***^ | 12.7 (1.5) |
| 18 mo | 13.5 (1.2)^**;a3,b3^ | 13 (1.3)^a3,b3^ | 12.4 (1.1)^e3^ | 12 (1.2)^e2^ | 12.4 (1.2)^**;f2^ | 11.6 (1.1)^f3^ | 13.5 (1.3) | 13.1 (1.3) | 13 (1.3)^***^ | 12.5 (1.4) |
| 24-mo | 13 (1.2)^***;b3^ | 12.5 (1.1)^a1,b3^ | 12.6 (1.2)^***^ | 12 (1.1) | 12.1 (1.1)^**^ | 11.5 (1)^f2^ | 12.5 (1.2) | 12.3 (1) | 12.6 (1.2)^***^ | 12.1 (1.1) |
| v) FM % |  | | | | | | | | | |
| 3 mo | 17.8 (6.1)^b3,c2^ | 18.5 (7.4)^b3^ | 18.2 (6.2)^*;d3,e3^ | 19.9 (7.9)^d3^ | 26.0 (9.0)^f3^ | 27.7 (11)^f3^ | 14.9 (5.3)^*^ | 17.9 (7.1) | 19.4 (8.1)^*^ | 20.8 (9.3) |
| 6-mo | 23.1 (7.0)^b2,c3^ | 24.3 (7.7)^b2,c3^ | 21.3 (6.4)^***;d3,e1^ | 25.0 (7.9)^d3,e1^ | 26.3 (8.1)^**;f3^ | 29.7 (9.6)^f3^ | 18.5 (5.9) | 19.5 (6.3) | 22.5 (7.4)^***^ | 24.8 (8.7) |
| 9 mo | 20.7 (7.0)^a1,b3,c2^ | 22.5 (6.9)^a1,b3,c2^ | 18.1 (5.9)^***;d3^ | 21.6 (6.4)^d3^ | 28.8 (9.4)^f3^ | 30.2 (8.9)^f3^ | 16.8 (5.7) | 17.4 (5.5) | 21.2 (8.5)^***^ | 23.3 (8.4) |
| 12 mo | 19.1 (6.4)^*;b3^ | 21.4 (7.0)^b3^ | 18.1 (5.8)^**;d3^ | 20.9 (6.8)^d3^ | 26.4 (8.5)^f3^ | 27.2 (8.8)^f3^ | 16.4 (5.6) | 16.7 (5.6) | 21.1 (8.3)^*^ | 22.4 (8.4) |
| 18 mo | 19.3 (6.9)^b3,c3^ | 19.9 (6.6)^b3,c3^ | 18.7 (6.7)^d3,e3^ | 20.8 (6.9)^d3,e3^ | 24.7 (9.0)^*;f3^ | 28.2 (9.3)^f3^ | 12.1 (4.5) | 12.0 (4.0) | 19.1 (8.2)^*^ | 20.5 (8.6) |
| 24-mo | 19.5 (5.5)^*;a1,b3,c3^ | 21.5 (5.5)^a1,b3,c3^ | 17.1 (5.0)^***;d3^ | 20.4 (5.3)^d3^ | 24.9 (7.0)^*;f3^ | 27.5 (7.0)^f3^ | 14.7 (4.2) | 14.9 (3.8) | 19.6 (6.8)^***^ | 22.0 (6.9) |

**^†^**a=Brazil vs Pakistan; b=Brazil vs South Africa; c=Brazil vs Sri Lanka; d=Pakistan vs South Africa; e=Pakistan vs Sri Lanka, f=South Africa vs Sri Lanka

*p<0.05, **p<0.01, ***p<0.001

^1^p<0.05, ^2^p<0.01, ^3^p<0.001
